# The Nursing Supplement

**Published:** 1888-09-15

**Authors:** 


### The Nursing Mirror.

All Contributions for this Supplement should be addressed "The Nursing Mirror," care of The Editor, 38, Old Jewry, E.C.

### En Passant.

AN AMERICAN LADY DOCTOR.—Dr. Mary Walker threatens to pay a visit to this country shortly to inaugurate her dress reform. For more than fifteen years Dr. Mary Walker has been carrying on her "dress reform." Her attire has been evolved out of many stages. At first she wore a loose grey dress with Bloomers. These gave way to silken trousers edged with lace. Now her usual dress is dark trousers, a morning coat, and silk hat. She wears a stand-up collar of the latest style, and a breastpin.

"FOR THY SAKE."—The following beautiful lines by Miss Christina Rossetti ought to prove encouraging to many of our readers:—

"Love Me in sinners and in saints
In each who needs or faints."—
Lord, I will love Thee as I can
In every brother man.

"All sore, all crippled, all who ache,
Tend all for My dear sake."—
All for Thy sake, Lord, I will see
In every sufferer—Thee.

GLASGOW ROYAL INFIRMARY.—It is seldom that such public praise falls to the share of the nurses as that which flooded the speeches at the opening of the new Home in connexion with the Glasgow Royal Infirmary. Mr. M'Ewen even went the length of making a touching reference to the death of Margaret Barrowman, a late nurse at the Infirmary, who, he said, had left a very fragrant name behind her. The proceedings were held in the covered corridor which connects the new Home with the main building, and there was a large attendance of ladies and gentlemen, besides those connected with the Infirmary.

ACCIDENTAL POISONING BY A NURSE.—A patient in the Belfast Royal Hospital, named James Jeffers, was accidently poisoned in that institution last week through taking a draught of carbolic acid which the nurse of the ward gave him in mistake for a black draught. After drinking a portion of the stuff, Jeffers remarked, "You have given me wrong medicine," and fell back insensible. The nurse having discovered her mistake by tasting the liquid, ran for the house physician, who was promptly in attendance. Antidotes were administered, but the man died an hour afterwards. The nurse (Miss Torrens), who was taken seriously ill after tasting the poison, and who still lies in a critical condition, has been placed under arrest. The affair has created quite a sensation in Belfast, where Miss Torrens' friends move in the best society.

WOMEN'S JUBILEE OFFERING.—We have received from the Countess of Strafford, the president of the General Committee of the Women's Jubilee Offering, the following: "I send you for insertion a letter which the Queen has been graciously pleased to write in acknowledgment of the jewel presented to her Majesty on behalf of the contributors to the Women's Jubilee Offering, at Osborne, on Tuesday, July 31."

Copy of the Queen's letter to Lady Strafford.

"Osborne, Aug. 13, 1888.

"Once more am I anxious to express to the women of Great Britain and Ireland and of the colonies, my warmest and most heartfelt thanks for the very handsome present they have given me, in recollection of my Jubilee. I shall wear with pride and pleasure the necklace and earrings which they have so kindly given me, as a precious token of their affection and loyalty."

OVERHEARD IN THE TRAIN.—"My dear, he was getting very old, and then he was so indecent I was obliged to get a trained nurse; he wasn't fit to be with anyone else!"

THE BAD NURSE.—She talks in a loud voice about her cases, and criticizes the physician's treatment. She tastes the beef-tea with a spoon, and puts it back in the broth, and washes dishes and clothes in the bath. She stands with her hands on her hips, and uses slang terms, such as "I guess," and "It was kind of middling," so that the house-physician can get no absolute facts from her. Unsightly vessels are in view all over her ward, and there are several wet circles on the tables where basins have been slopped down. The bedclothes on all the beds are crooked and stained with food and medicine. Her dress is stuck all over with pins, and she sits down at intervals on the nearest bed to tie up her shoe-strings. In feeding a helpless patient, she never tucks a towel under his chin; and uses a large spoon so rapidly that the food runs out at each corner of the man's mouth. Her flowers are stuck anyhow into broken vases full of dark, smelling water, and she uses lint to clean up the messes she is always making. She is good-humoured, but forgetful, and if convalescent patients like her, those who are really ill loathe her, her fellow-nurses scorn her, and those in authority long to get rid of her.

STRANGE DEATH OF A CLERGYMAN.—An inquest was held last week at Twickenham on the body of the Rev. F. D. Eyre, a clergyman of the Church of England, aged eighty-six, whose body had been found to be extensively bruised after death. The nephew of the deceased gentleman said he had paid a nurse named Davis £3 or £4 a-week to attend to him, but was not satisfied with Davis's behaviour. Miss Schilling, of Twickenham Common, gave evidence to the effect that she had been frequently called to the house of the deceased gentleman by Mrs. Davis, because he would not let the latter prepare food for him. He had said he was afraid that she would poison him. On August 19th, he said she had been "going on" at him, and, in the witness's presence, she then stripped the bed, seized him by the feet, and pulled him on the the floor. The witness steadied him, and she did not think he was bruised on that occasion. Dr. Ward, police divisional surgeon, Twickenham, said he was privately called to attend the deceased on July 13th. The latter abused him in unmeasured language, and for some time the witness could do nothing with him. He was suffering from dropsy, and was so dirty that the witness threatened to scrub him. This threat had a good effect. The witness, however, believed that the medicine he supplied was never taken. Finding that he could not do the patient any good under the circumstances, the witness eventually said he should not attend again unless he was sent for. He was again called in on the 3rd inst., and Mr. Eyre died the same night. The external marks on the body included a scalp-wound about half an inch long over the left eye, several bruises on the left side of the face and neck, and numerous marks of the same character on the back of the neck and between the shoulders, while there was a partially healed wound over a rib on the right side, and other bruises on the elbows and wrists. The cause of death was general want of nutrition; but death might have been accelerated by anything that tended to weaken the deceased, and witness thought that the injuries must have done so. Many of the bruises might have been caused by his falling about. The inquest was then adjourned for the evidence of Mrs. Davis and other witnesses.

### Original Articles.

### A SISTER'S HOLIDAY.—Part I.

It was with a delightful feeling of putting aside all cares and responsibilities that I folded up my long blue gown and aprons and put on a grey travelling suit instead. For a whole three weeks I was to be off duty, and, far from the worries of ward management, and far from London smoke, I was to take my ease and enjoy a well-earned holiday. The bustling excitement at the station acted as a stimulant to my weary mind, and after the train first started I eagerly watched the country scenery for awhile. How green, and fresh, and quiet, and comforting the country looked! And then, before I understood it, I was sleeping peacefully in my corner of the carriage, while the train whirled onwards towards its northern destination. I was lucky in having had an invitation to spend my holiday with some dear old friends who lived by the sea, and as I had never visited them before, nor ever been on that part of the coast, I had a thorough change before me. For the first week I spent my time in lying idly about on the beach, or wandering about the garden. My friends were great tennis-players, but I had not vigour or practice enough to engage with them in that game.

"What is that big building over there?" I asked my hostess one morning, as we sat looking across the bay.

"Oh, I believe it's the infirmary."

"What a splendid position; it must be hospital and convalescent home in one. Who is the matron?"

"She is called Miss James. I don't know anything about her."

I thought I did, for one of the sisters I had trained under was a Miss James, and I longed to find out if it was the same person. A few days afterwards I found myself just outside the infirmary gates, and on the impulse of the moment I turned in and asked to see Miss James. I recognised my old friend at once, and we had a long chat over the past together. For ten days I had never opened my mouth on hospital subjects, and it was a real relief to find myself in the company of a fellow-nurse once more. It is all very well for people to write that a nurse should forget her profession altogether during her holidays, but I believe it is a physical impossibility. When for nine months of the year all the forces of the mind are concentrated on one subject, it is not so easy for the brain to forget its habitual train of thought. There is a celebrated oculist who, when travelling for rest and recreation, cannot help noticing the eyes of his fellow-travellers. Sooner or later he discovers someone on the verge of blindness for want of an operation. Then the oculist gets restless and unhappy; he tries to go on his way and forget, but he cannot. At last, in a fit of desperation, he seeks the sufferer, and gives him his opinion, and undertakes a cure. The patient is grateful, the oculist happy and cheerful; the operation is performed, and, with expressions of mutual gratitude, doctor and patient part. The oculist's friends say he simply travels to seek new subjects for his skill, but it is not so; he has had strength of mind enough to start without his instruments on some occasions, but has always had to telegraph for them. It is a simple fact that he is so bound up in his work that he never forgets it. Well, in the same way, I, who had looked forward to being absolutely free from all thoughts of nursing, found the keenest pleasure in hearing of Miss James's trials and difficulties in her out-of-the-way post. Tea came in, the sun went down, and if a bell hadn't announced that it was prayer-time we should have gone on talking yet longer. I was late for dinner, and was quite ashamed to confess to my friends that I had spent the afternoon at the infirmary. The next day I walked determinedly out into the country, and threw myself down under a tree in a lonely wood. I fingered the blades of grass, and tried to recall the names of the flowers. Not many years ago I could spend hours of perfect happiness in merely lying listening to the birds and watching the clouds float over the sky. To be alone with Nature was at that time my idea of bliss, and I was content to be day after day engaged in observing her ways. Every leaf and insect interested me, and I had a pleasant feeling that I was in touch with Nature, and could sympathise with all her pleasure and pain. Now I was uncomfortable beneath my tree: the insects annoyed me, the flowers did not interest me; do what I would, my restless thoughts would turn towards hospital matters. I remembered Miss James's remark when I told her news of all of whom she asked—"You are certainly in touch with all the nursing world." Ah, yes! there was the difficulty; I had lost my love of nature in my love of my work. The thought saddened me for awhile, and I tried once more to concern myself with the woodland world, and to enjoy its calm and beauty. It was no use, and reluctantly I gave up the attempt and returned towards the small seaside town. My steps grew more rapid as I approached the bay, my eyes sought the large building on the cliff opposite, and I determined to ask Miss James to let me go round the wards and see all the arrangements. I tendered my request that very afternoon, and it was willingly complied with.

"We are going to have great alterations next spring; the building is to be enlarged, and we hope to get several new conveniences for the wards. I should be very glad for any hints concerning the latest improvements in ward furniture. You see it is five years since I left London, and I don't doubt we are a bit old-fashioned."

We started for a wander round the wards at once, and I got a general idea of the infirmary arrangements. There were four large wards of twelve beds each: two on the first floor for surgical cases, women on one side, men on the other; two on the second floor for medical cases, one for men and one for women. There were also four little wards, holding two beds each, and a fever pavilion in the grounds, which held ten beds. These, together with four cribs for small children, made a total of 70 beds, and the nursing staff for this fairly-large number of patients was so absurdly small, that I simply refuse to tell it. If our matron knew how few women managed the entire nursing of that infirmary, and managed it uncommonly well, too, she would consider myself and fellow-nurses as lazy beings, so I shall simply keep silence about the wondrous working powers of our northern sisters. The wards seemed very quiet, for there were no doctors about anywhere, and I inquired as to the medical staff.

"Our house surgeon lives in that little building there, and gives us all his time. I go round with him every morning," said Miss James, "and sometimes do a few dressings for him, and I always attend at operations. We have two visiting physicians and two surgeons, and they attend alternate months. The house surgeon does the dispensing and keeps the case-book, for our cases are not fully entered on the bed-head cards, nor do we ever write there what the patient is suffering from."

"There are no students, I suppose?"

"Sometimes students who are staying or living here walk the wards regularly for a time; and when there is an operation of any importance, or rather, an afternoon of operations, we generally have quite a concourse of doctors and students. We get very good cases here. I'll tell you what, you come round with Dr. Evans to-morrow morning, and he'll show you all his dressings, and explain the cases."

"Oh, no! I couldn't do that, I have no uniform with me."

"That doesn't matter; the doctor is my very good friend, and always glad to have a companion on his rounds. Come, now, I shall expect you at ten to-morrow morning."

Well, I hadn't seen much surgical nursing since I had become a sister, and there is always something new to be learnt from every case; so I gladly accepted this kind offer, and went home quite elated at the prospect of increasing my nursing knowledge on the morrow.

*(To be continued.)*

### Letters from a Probationer.

No. III.—THE HOSPITAL CHAPEL.

DEAR ———, This is the Sabbath day, and I have just been to chapel, where I was much impressed by the sermon. The chapel is a very pretty little building in the grounds, which you reach by a covered passage, and on Sundays constant short services are held there, which all nurses are supposed to attend. The scene in the chapel is very strange, for all the patients who are well enough come in the red cloaks provided by the hospital, and sit in the front seats; the women on one side, the men on the other. The aisle is generally full of wheel chairs or reclining couches, in which there are other patients not quite so well. Behind the patients sit the nurses all in uniform—print dresses and clean white caps and aprons. Behind them again sit the sisters, in their dark dresses and more elaborate caps; then the scrubbers and porters, each in their uniform, and in the far back-ground the matron, and perhaps a house-surgeon or visitor or two. Sunday is rather a hard day on the nurses here, for the sisters are often out, and the only time we get off duty is an hour to go to chapel. The service is very well conducted though the choir is rather meagre and the organ very wheezy, but the charm to-day was the presence of a strange preacher, who instead of addressing the patients spoke chiefly to the nursing staff. Of course, the patients are the first consideration in a hospital, and though I have heard many sermons here, I never before heard one not specially preached for them. But our minister to-day took for his text "Be not weary in well doing," and spoke with a wondrous conception of the weariness which often falls to a nurse's lot, till one could almost imagine he had suffered from the swollen feet, aching back, and heavy head which frequently accompany us to our beds. It was a very encouraging and sympathetic sermon, but all the time the context was ringing in my ears —"For in due time ye shall reap, if ye faint not."

Ah, yes! if ye faint not, but how many there are to whom the harvest never comes, and who faint long ere the reaping time! I wonder did this clergyman know that there are always three or four of our nurses "warded," *i.e.*, occupying beds in the wards as patients, and that this year a week has seldom gone by without the ambulance from the Fever Hospital coming to carry off a nurse. I am afraid we often get weary all round—weary in body and mind, and weary in well doing also. The nurses' ideal is a fearfully high one to strive after, and it requires enormous moral strength and courage not to faint at times. I know I was feeling very cross and depressed, and ready to snap anyone who spoke to me when I went to chapel this morning. The service soothed me a little, and made me ashamed of myself also, and then when I found that the sermon was for me and my fellow nurses, that it was to point out our temptations and help us to fight them, I was quite cheered and invigorated. A little sympathy and a few kind words go a long way, and I am sure many of us returned to the wards with a firm determination not to give way to weariness in well doing at least. There are often strange interruptions to the service held in the middle of sick beds like this: sometimes it is a patient falls back in a faint or a fit, and has to be carried out; or a nurse comes swiftly and silently up the aisle to some sister, to whom she whispers a message, the import of which we all guess too well. Surely no mission service in a far-off land presents a stranger sight than our little chapel with its rows of women in different uniforms. I am sure if the services were open to the public, many would be touched by the spectacle and their compassion roused for those who worship here. When we have one of the simple old pathetic hymns, such as "Abide with me," it is often too much for many of the patients, who are weakened by illness. The week-day services in the wards never seem to have the same effect; this may be because the nurses are bound to move about a little, and there is much to distract the attention. I hope you may attend our chapel some day.

### Glasgow Royal Infirmary.

### OPENING OF NEW NURSES' HOME.

ON August 31st the new Nurses' Home in connexion with the Glasgow Royal Infirmary was formally opened. It is situated a little off the east of the main block of the institution, and it is believed to be the most perfect of the kind in the kingdom. Four storeys in height, it contains a large recreation hall and other public apartments, as well as 85 bedrooms. It was erected at a cost of £8,000.

Mr. HUGH BROWN, Chairman of the House and Building Committee, made a most interesting opening speech, in which he said that it was not necessary that he should take up their time by giving a minute description of the Home, as they had had an opportunity of inspecting it, and a very full description of it had appeared in the newspapers. It could accommodate 82 nurses, so that they had now altogether single rooms, unconnected with wards, for 120 nurses. In the erection and furnishing of the Home the managers had endeavoured to avoid on the one hand extravagance and unnecessary expense, and on the other to secure brightness and comfort, and so make it in reality, as well as in name, a home. As soon as it was occupied, forty beds would be liberated in the hospital, which would be available for patients, and the old recreation-room might also be used for some other purpose, and so be of much advantage. He had still to explain why the elegant and spacious erection in which they were now assembled came to be put up. Whilst it was desirable, and, in fact, necessary, that the Home should be at some distance from the hospital, it was equally necessary that the nurses, in coming and going to and from it, often in wintry and tempestuous weather, should not run the risk of getting cold, and so, by becoming patients themselves, neutralize the expected benefit. It was therefore resolved to provide a covered passage, and it was found that for a proportionately small increase of cost it could be made of such dimensions as to be a valuable accessory to the hospital as a place where convalescent patients might, without risk, get some exercise. Many friends had promised to send plants, and they hoped before long that it might have something of the aspect of a winter garden. The question of hospital management and accommodation was one that in a city like this engaged, and properly so, much attention. It was not for him to say anything on the subject, but he might remind those there who did not know that this was the second largest hospital in Scotland that it contained 580 beds, that they had a staff of 187, and a resident population within its walls of 700 people, and that more accident cases were treated than in any other hospital in the kingdom. They might thus understand that the managers, and more especially the administrative staff, had a serious responsibility thrown on them, and he was sure the meeting would approve of the course that the managers had followed in doing what they could to increase the comfort and happiness of those who had not the least arduous part of the duty to perform—the nurses—by the erection of that Home.

Dr. DUNLOP, who was next called upon, said he was exceedingly sorry that Dr. M'Ewen was not present that day to make the observations which were expected of him. He (Dr. Dunlop), wished to say just in a sentence, as one of the oldest surgeons of the institution, that they benefited very greatly by the new style of nurses which they had, as compared with the nurses which were common in the house 25 years ago. They had now a highly-educated class of young women, who were able to bring to the discharge of their duties high intellectual qualities, combined at the same time with a very great amount of zeal and earnestness, and a willingness to do actual work. Their duties were not by any means of an ornamental kind. They were eminently practical, and he thought they were eminently useful. The great success which had attended the work of the institution in the medical wards, and especially in the surgical wards—of which he had personal experience—had been mainly due to the great care and to the experience of the cultivated ladies who were now their nurses; and the public were indebted to them to a large extent for the low mortality at the infirmary.

Mr. M'EWEN then made a short speech, in which he said the nursing staff of the Royal Infirmary he looked upon as efficient as any—and he had visited all the large hospitals in the country. From a list he had got made up, he found they had about twenty nurses, who had been upon an average twenty years in the hospital. He did not think they would get any establishment in which such a number had all that time maintained an unblemished character and acquired distinction in the profession they had adopted. He had always taken a great interest in the staff, and he was happy to say that about twenty connected with the hospital had been removed to other hospitals, where they now occupied very important spheres. The new building, he thought, was perfect.

The LORD PROVOST (Sir James King, Bart.) then declared the Nurses' Home open, and the proceedings terminated with much applause.

### Everybody's Opinion.

[*Correspondence on all subjects is invited. No communications can be entertained if the name and address of the correspondent is not given, or unless one side of the paper only be written on.*]

### THE RED CROSS.

I AM writing in something of a dilemma. There is awaiting erection a signboard of most *recherché* design, indicating the exact locality of this institute. It has a dark-blue ground with red letters, and each side of the word "Nurses" is the red cross. Will you kindly inform me through your columns, whether I am entitled to have this emblem on the board? It has recently been suggested to me that I have not.

Tunbridge Wells. JESSIE MURRAY, Matron.

[The red cross belongs to the Red Cross Society for Nursing the Sick in Time of War; but there is no objection to its being used for the Tunbridge Wells Nurses' Institute, as it is not a trade mark nor a special badge.—ED. *T. H.*]

### ZENANA MEDICAL COLLEGE.

I HAVE no desire to enter into any argument on the much-debated question of the two years' training. In giving the training we but put a power into the hands of the students, to be increased and developed more and more by practical work. This is where people make a mistake, they think that when children leave school their training is complete, and talk of such as having "finished their education," when the truth is only the foundation has been laid, the building has yet to be erected. The same applies to medical training. Then too much depends on the students themselves. What one may acquire with ease in two years, or even in one, may never be learnt by another, though a lifetime be devoted to it. I remember a well-known physician remarking to me on one occasion that he felt as if sanctified common sense did far more for a nurse than the ordinary training of such. I fancy the old saying "actions speak louder than words," will hold good with reference to this subject. We have positive proof in the testimony that comes from abroad of the efficient work done; and having myself been treated in India by some of the students from the college, when it seemed to me that life trembled in the balance, I have reason to testify to the value of the training given here. One word more. So different are the climate and surroundings of foreign countries, that ladies trained in England have to lay aside many preconceived ideas, and adapt their treatment to the customs and prejudices of those with whom they are brought in contact, and for whom they have to prescribe.

MARY A. ROBINSON (Zenana Missionary).

### Appointments.

TIVERTON INFIRMARY.—Miss Margaret O'Reilly has been appointed matron of this Infirmary in place of her sister, who has resigned. Miss Margaret O'Reilly was trained at the Nurses' Training School, Liverpool, and for the past six years filled the post of head nurse in the Devon and Exeter Hospital. For the space of one month Miss O'Reilly acted as deputy matron at Tiverton. Miss O'Reilly's testimonials from both physicians and surgeons at Exeter are of the very highest order, and all of them seem to regret her departure. But the most remarkable testimonial amongst Miss O'Reilly's number is one from the Chaplain of Exeter Hospital, who simply does not seem to know how to say enough in her favour. We congratulate Tiverton on its new matron.

### Honours and Presentations.

MISS O'REILLY, the late matron of the Tiverton Infirmary and Dispensary, has, on the occasion of her marriage to Mr. Allnutt, been presented with a service of plate with a suitable inscription from the Committee, the cost of which has been met by private subscription. Mrs. Allnutt is greatly pleased with this proof that her services have been appreciated.

### Lectures.

A SERIES of lectures is to be given at the Midwives' Institute and Trained Nurses' Club, the first of which will be delivered on Friday evening, September 21st, at seven o'clock. Subject—The Responsibilities attaching to the Midwifery Nurse during Pregnancy. Admission to members' friends, 6*d.*

THE sixth course of lectures for sanitary inspectors commences on September 25th at the Parkes Museum. The introductory lecture will be given by Dr. George Vivian Poore, F.R.C.P., on "General History, Principles, and Methods of Hygiene," commencing at eight p.m. Fee, 5*s.* for the course.

### Vacancies.

The following vacancies are announced:—

**Sister.**

Borough Fever Hospital, Leeds; £30.

**Nurses.**

Bradford Eye and Ear Hospital; £25.

Kent Nursing Institution.

Southport Trained Nurses' Home.

Canterbury Institute; several.

Kendal Hospital; £28.

Sheffield General Infirmary; £21.

Sheffield Nurses' Home; £25.

Victoria Home, Bournemouth.

County of Cornwall Trained Nurses' Home, Truro; two.

Sheffield General Infirmary; several.

Borough Fever Hospital, Leicester; £20.

General Infirmary, Hertford; (Head), £25.

West Derby Union, Liverpool; £25; two.

Northampton Institute.

Nottingham Institution; £25.

Radcliffe Infirmary; two.

Kingston Nurses' Home, Hull; several.

Hospital for Paralysis and Epilepsy, Regent's Park; £20.

The Infirmary, Rochdale; £22.

Nurses' Institute, Canterbury; two.

Ipswich Nurses' Home; three, £22.

Home for Trained Nurses, Bath; two, £25.

Maud Hospital, Exmouth: (visiting), £30.

**District Nurses.**

Carrickfergus.
Beaumaris.

**Probationers.**

Royal Hospital for Diseases of the Chest.

Batley Cottage Hospital.

Lowestoft Hospital.

Lloyd Cottage Hospital, Bridlington.

**Laundry Woman.**

Female Hospital, Woolwich; £1 per week.

### Notes and Queries.

TO CORRESPONDENTS.—1. Questions may be written on post-cards. 2. Advertisements in disguise are inadmissible. 3. In answering a query please quote the number. 4. If a private answer is desired, a stamped addressed envelope must be forwarded.

**Queries.**

(56) *Linen Cuffs.*—Sister R. would be glad to know where the deep linen cuffs can be bought like the Netley sisters wear?

(57) *Military Hospitals.*—Do military hospitals take probationers, if so, to whom should I apply to become one at the Herbert Hospital? *C. L. V.*

**Answers.**

(44) Would Nurse A. like to get a dozen turn-down collars, and sell Sister R. the half-dozen; as surely a nurse need only have six?

(54) *Nurse D.* can get training in massage at Mrs. Holder's, West Street, Brighton. Terms, three guineas.

(59) *Pericardium.*—If you are a medical man, and will send in your card at the London Hospital Medical School, you will be admitted.

(60) *Lethe.*—The last article on nursing which appeared in "The Lady" was in the number for January 19, 1888.

(61) *Bella* should address Mrs. Nicholls, secretary of the Midwives' Institute and Trained Nurses' Club, 15, Buckingham Street, Strand, W.C.

